# Identifying Priority Giant Anteater (*Myrmecophaga tridactyla*) Populations for Conservation in São Paulo State, Brazil

**DOI:** 10.1002/ece3.6809

**Published:** 2020-11-18

**Authors:** Ricardo Quiterio Sartori, Alessandro Garcia Lopes, Luiz Paulo Nogueira Aires, Rita de Cassia Bianchi, Cinara Cássia Brandão de Mattos, Adriana Coletto Morales, Lilian Castiglioni

**Affiliations:** ^1^ School of Biosciences, Humanities, and Exact Sciences Graduate Program in Biosciences São Paulo State University (UNESP) São José do Rio Preto São Paulo State Brazil; ^2^ School of Agricultural and Veterinary Science São Paulo State University (UNESP) Jaboticabal São Paulo State Brazil; ^3^ São José do Rio Preto Medical School (FAMERP) São José do Rio Preto São Paulo State Brazil; ^4^ Rio Preto University Center (UNIRP) São José do Rio Preto São Paulo State Brazil

**Keywords:** Brazil, conservation genetics, giant anteater, microsatellite markers, population structure, São Paulo

## Abstract

Habitat loss is the main threat to biodiversity conservation worldwide. Some species may be particularly susceptible to the effects of fragmentation and the isolation of populations. The impacts of human activity on wild animal populations may be understood through relationships between individual genetic data and spatial landscape variables, particularly when considering local population dynamics influenced by fragmented habitats. Thus, the objective of this study was to analyze the population structure and genetic diversity of the giant anteater (*Myrmecophaga tridactyla*) using an individual sampling scheme (ISS) on a regional geographic scale. Data were collected from 41 specimens from twenty different locations in São Paulo State, Brazil, and six polymorphic microsatellite loci were genotyped. Our results indicate that barriers to gene flow exist and have segregated individuals of the farther away areas into two spatially structured clusters. The populations were also found to have high genetic diversity. The experimental sampling approach used herein enabled an analysis of the population dynamics of the giant anteater on a regional scale, as well as the identification of priority populations for genetic resource conservation for this species. The results reflect the need for adequate management plans. The efficacy of the sampling scheme may vary based on the study model used, but we argue that the use of an ISS combined with suitable molecular markers and statistical methods may serve as an important tool for initial analyses of threatened or vulnerable species, particularly in anthropized regions where populations are small or hard to characterize.

## Graphical abstract

Habitat loss is the main threat to biodiversity conservation worldwide, and some species may be particularly susceptible to the effects of fragmentation and the isolation of populations. Thus, the objective of this study was to analyze the population structure and genetic diversity of the giant anteater (*Myrmecophaga tridactyla*) using an individual sampling scheme (ISS) on a regional geographic scale in São Paulo State, Brazil. Our results indicate that barriers to gene flow exist, and the populations present high genetic diversity, which reflects the importance of these populations and the necessity of immediate conservation actions.

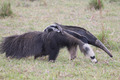

## 1 INTRODUCTION

Decreases in and losses of habitat connectivity lead to increases in the existing decline in wild animal populations, thus raising the risk of their extinction (Butchart et al., 2010). The transformation of a continuous habitat into “small pieces of habitat” may affect biodiversity at the population level, with changes in species number, distribution, reproduction, and survival (Carvalho et al., 2009; Fahrig, 2003; Rocha et al., 2018; Wolff et al., 1997).

**Figure 1:**
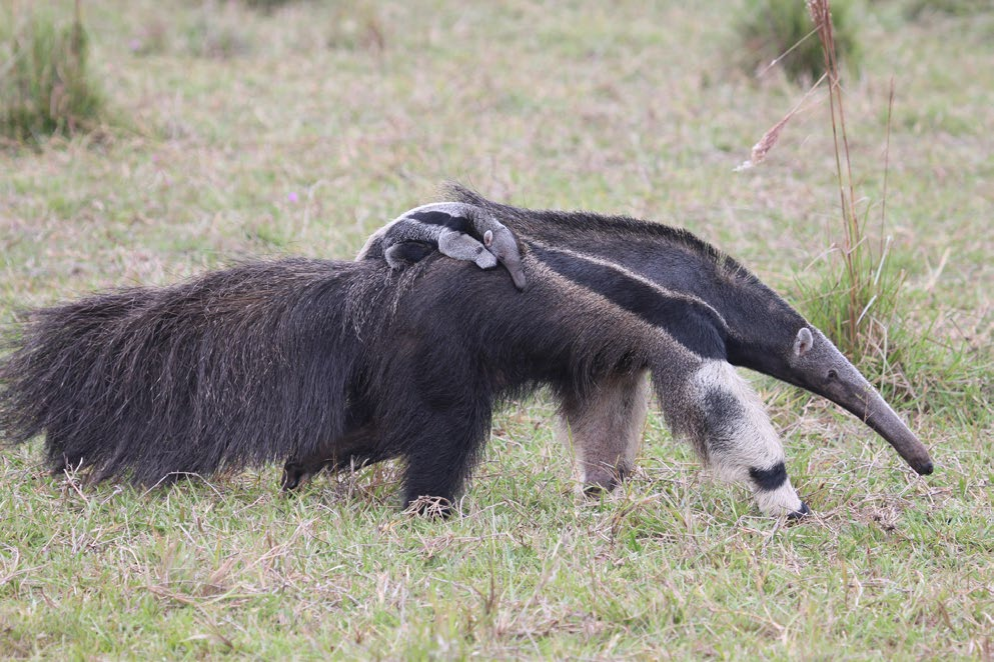
The giant anteater, *Myrmecophaga tridactyla* (Pilosa: Myrmecophagidae). Photo credit: Jason Woolgar

Medium‐ and large‐sized land mammals may be particularly vulnerable to habitat fragmentation and loss, particularly for species with large home ranges or long life cycles (Keinath et al., 2017; Morris et al., 2008). A large‐sized animal averaging 31 kg, the giant anteater (*Myrmecophaga tridactyla*; Linnaeus, 1758) (Figure 1), is considered a vulnerable species in Brazil largely because of its habitat loss in the Cerrado and other Brazilian biomes (Miranda et al., 2015).

There is a tendency for the remaining populations of *M. tridactyla* to become progressively more isolated, which, in turn, further increases their vulnerability and leads to various cases of local extinction. Some of the species’ biological characteristics may lead to low rates of population growth: a low metabolic rate and low thermoregulatory capacity, limited reproductive potential, prolonged parental care, a long gestation period, and solitary habits (Clozato et al., 2017). The species is a frequent victim of roadkills, as well as of fires caused by sugar cane crop burning and prolonged periods of drought. These factors threaten the persistence of giant anteater populations in anthropized areas (Diniz & Brito, 2013, 2015).

The extinction of small populations is expected in many fragments over time; the study of local population dynamics influenced by the existence of highly fragmented habitat networks may provide clearer information that can be used to implement strategies to help protect and manage isolated populations (Canale et al., 2012; Weiss & Leese, 2016).

Conservation genetics focuses on the effects of contemporary genetic structuring on long‐term survival of a species, and genetic analyses have been used to determine the prospective status of several endangered species (Wan et al., 2004). This study sought to characterize the giant anteater's population structure in fragments of Cerrado and Cerrado/Atlantic forest ecotones, to evaluate its genetic diversity, to detect any barriers to gene flow, and to determine the current degree of gene flow in anthropized areas in São Paulo State, Brazil.

## 2 MATERIALS AND METHODS

### 2.1 Study area

The animals sampled herein were largely distributed in the northwestern and central‐southern region of São Paulo State, which is the most industrialized state in Brazil. This region is currently experiencing intense expansion and urban development, with native vegetation being highly affected by human activity (Durigan et al., 2007). Before colonization, this region was covered by a mosaic of Cerrado and seasonal semi‐deciduous forest; however, the forests have since been almost entirely depleted (Myers et al., 2000). The vegetation cover of São Paulo State has been drastically reduced, and the remnants correspond to small areas surrounded by pasture, sugarcane, soybean, reforestation, perennial crops, and urban zones (Kronka et al., 2005).

The study area included some animals that had been obtained, most frequently after car accidents, in cities that border the Noroeste Paulista Conservation Area (EENP) located in the cities of São José do Rio Preto and Mirassol. Other animals were obtained in search‐and‐capture campaigns in and around the Santa Bárbara Conservation Area (EESB) located in the city of Águas de Santa Bárbara. The use of animals from these locations permitted inferences about the status of individuals and populations in habitats in and around these conservation areas.

The EENP conservation area is 500 ha in size when combined with the Noroeste Paulista State Forest and is considered to be seriously threatened by urbanization and real estate speculation. It is surrounded by pastures, farms, and urban neighborhoods (IF – Instituto Florestal, 2014). The EESB conservation area is 2,712 ha in size, and most of the area is occupied by native vegetation. There is little evidence of human disturbance, and the area is surrounded by pastures and reforestation sites (IF – Instituto Florestal, 2011).

### 2.2 Sampling

The current study included a local analysis involving an ISS—individual sampling scheme (Prunier et al., 2013). The ISS used allowed for giant anteaters in the northwestern and central‐southern regions of São Paulo State to be analyzed.

The samples included in this study (*n* = 41) were obtained from different sources. Some were collected by veterinarians and park rangers after the collection of animals that had been run over or had been victims of crop burning (*n* = 35). In other cases, blood samples were obtained from animals captured alive in field studies for other purposes and were provided to our laboratory (*n* = 6).

**Figure 2:**
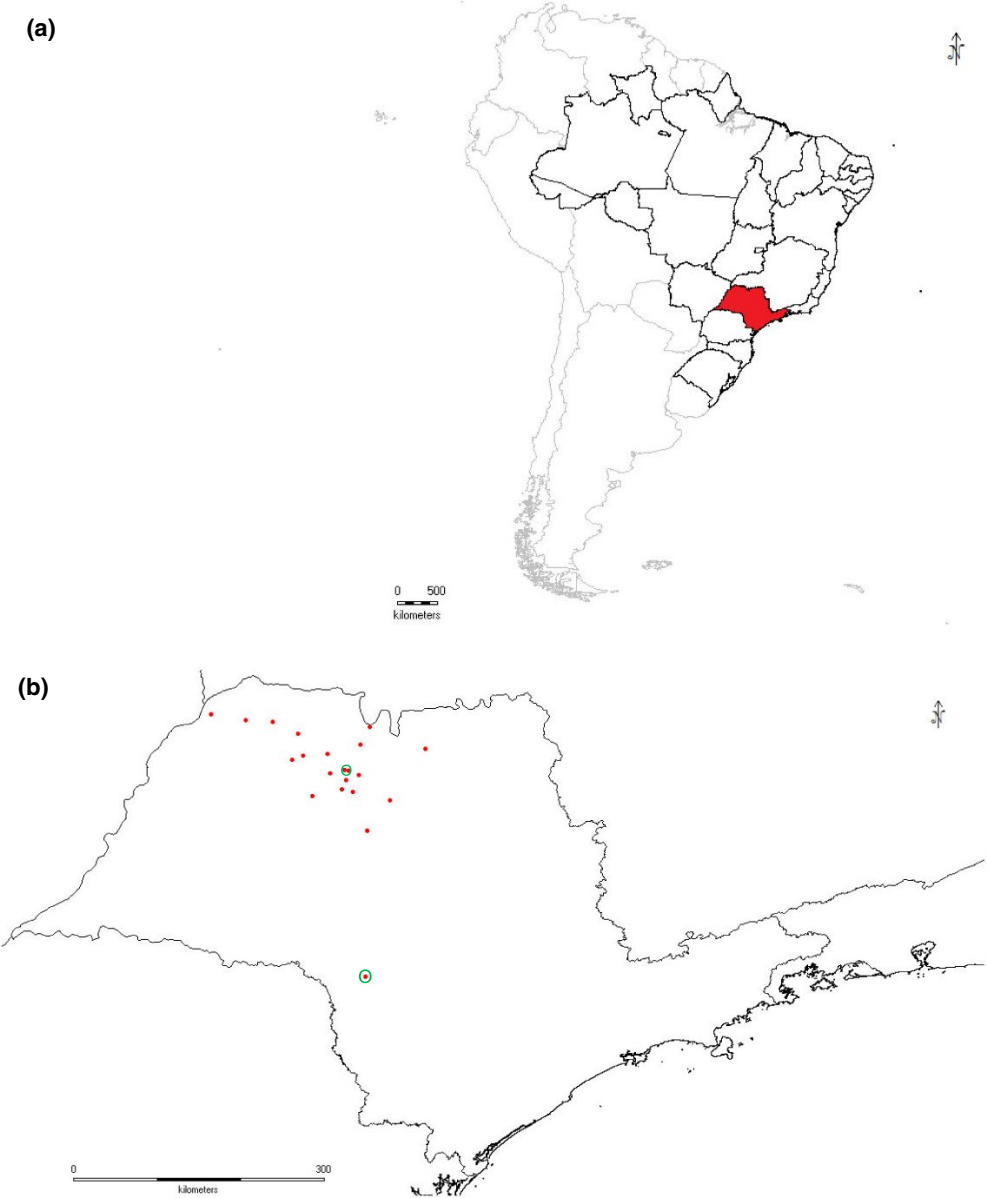
(a) Map of the study area (São Paulo State, Brazil). (b) Red dots represent the sampling points, and green circles represent the conservation areas

Samples were obtained from a total of twenty different sites. The geographic distances between the sampling points ranged from 10 km to 340 km (Figure 2). The maps were generated in DIVA‐GIS, version 7.5 (Hijmans et al., 2012).

The analyses of interindividual information used in this study may be applied on practically any spatial scale, including in analyses of genetic data that use one or several individuals from different sampling sites (Guillot et al., 2009; Mank & Avise, 2004; Miller, 2005; Safner et al., 2011). The number of individuals required to evaluate genetic structure depends on the extent of population differentiation, as well as on the degree of polymorphism in the markers; these factors mean that a small number of sample animals may be appropriate in certain circumstances (Landguth et al., 2012).

All biological material collection herein was authorized for scientific purposes by the Biodiversity Information and Authorization System (SISBIO) from the Brazilian Federal Environmental Agency (IBAMA) under certificate number 43854‐4 and was approved by the research ethics committees at the universities involved in this study (Rio Preto University Center [UNIRP] under authorization number 04/2014PP and São José do Rio Preto Medical School [FAMERP] under authorization number 2014/05302‐8). For the captures in the EESB conservation area (the samples provided from previous studies), sampling licenses were obtained (COTEC No. 429/2014 D23/2013 PGH; SISBIO No. 38326‐5), as was authorization from the local institutional animal care and use committees (CEUA No. 003414/13). Details on the origin of the samples, geographic coordinates, and genotypes are available in the Dryad Data Repository (https://doi.org/10.5061/dryad.cfxpnvx31).

### 2.3 Amplification conditions

Six pairs of microsatellite‐specific oligonucleotides were used in the molecular analyses, as detailed in Table 1 (Garcia et al., 2005). Genomic DNA was extracted using the QIAmp DNA kit (Qiagen) following the manufacturer's instructions. It was quantified using an Epoch™ Spectrophotometer System (BioTek) to measure DNA quality and concentration.

**Table 1:** Microsatellite‐specific oligonucleotides (Garcia et al., 2005)

Locus	Repeat motif	Primer sequences	T (°C)	Size (bp)
MtriUSP 04	(gt)09	5′GGGTCAGATATCCTAATGGG3′	61	158
		5′TGTCTTCTTTACTCAGTGCTCC3′		
MtriUSP 07	(gt)42	5′AGGAGGATAAGATTAGGCAG3′	62	274
		5′TGTGTCCTGTGAAGTAATGG3′		
MtriUSP 11	(gt)15	5′TGTCTCTGTGTTAGGGTTCTTC3′	59	174
		5′TCACCTTCATTGGAGCTTC3′		
MtriUSP 13	(gt)14	5′CTGCTCAGGTAACATTCC3′	57	223
		5′TGGTAAAGAATGAGGTC3′		
MtriUSP 17	(gt)20	5′CCCAGAATGAACTTACTTG3′	55	191
		5′ACTGGCAACTTGTTGTTG3′		
MtriUSP 20	(gt)14	5′CTTTCCTCATATCTCCCTG3′	55	157
		5′CTATATGCTTGCCTTTGG3′		

Amplification via PCR was performed with the addition of the M13 tail, which allowed for the use of the tailed primer method (Schuelke, 2000). The amplifications were performed using touchdown PCR in a total volume of 17.2 μl, which contained 1 μl of DNA (~50 ηg), 0.2 μl of the forward primer (5 µM), 0.4 µl of the M13 primer dyed with fluorophore (5 µ), 0.6 µl of the reverse primer (5 µM), 5 µl of the Gotaq^®^ Colorless Master Mix (Promega; 2X), and 10 µl of ultrapure water (Don et al., 1991; Korbie & Mattick, 2008).

The amplification conditions included denaturation at 95°C for 2 min, 12 cycles at 95°C for 1 min, between 64°C and 52°C for 40 s (−1°C per cycle), 72°C for 30 s, plus 25 additional cycles (1 min at 95°C, 40 s at 52°C, and 30 s at 72°C), and the final extension for 5 min at 72°C. After the amplifications were confirmed in 3% agarose gel, the fragments were identified in an ABI 3500 Genetic Analyzer (Applied Biosystems). The DS‐33 GeneScan™ Installation Standard (Applied Biosystems) was used for the capillary electrophoresis run, and the reactions were organized into two groups based on the expected fragment sizes.

The results and peak sizes were analyzed using GeneMapper^®^, version 4.1 (Applied Biosystems, 2012). The correct identification of the alleles is crucial for a reliable interpretation of the microsatellite data (Arif et al., 2010). No artifacts or stutter peaks negatively affected the differentiation between homozygote and heterozygote, and the alleles exhibited well‐resolved peaks. The fragments were larger in size relative to the size of the original markers due to the addition of the use of the M13‐tailed primer, but the sizes corresponded to the expected sizes based on the information available on their development (Garcia et al., 2005).

### 2.4 Statistical analyses

GENEPOP Software, version 4.2 (Raymond & Rousset, 1995), was used to determine the presence of null alleles based on the maximum likelihood method, (Dempster et al., 1977). No genotyping failure was detected and the original dataset was used for the remaining analyses.

To characterize the population structure, individual‐based Bayesian analyses were employed using the programs STRUCTURE v.2.3.4 (Pritchard et al., 2000) and Geneland 4.0.5 (Guillot et al., 2005) to determine the most likely number of clusters (K).

In STRUCTURE, we ran 25 replicate runs for each potential number of genetic clusters with 100,000 burn‐in steps, 20,000 MCMC repetitions, and 25 iterations per run. Values of *K* = 1 to *K* = 5 were tested using the model with admixture, correlated allele frequencies, and the ancestry prior option “separate α for each population” (Falush et al., 2003). The results were then used in STRUCTURE HARVESTER (Earl & Vonholdt, 2012) to select the K value associated with the highest mean posterior probability of the data (Evanno et al., 2005) with postprocessing in the CLUMPAK program (Kopelman et al., 2015).

Unbalanced sampling has a large impact on STRUCTURE analysis. It reduces the quality of individual assignments to populations and the accuracy of the estimated number of populations. However, these adverse effects of unbalanced sampling on Structure analysis can be largely overcome by simply switching to the alternative ancestry prior, at least when the number of populations is not large and STRUCTURE can yield highly accurate inferences of individual ancestries (Wang, 2017).

Population structure was also analyzed with the software GENELAND 4.0.5 (Guillot et al., 2005) and R version 3.4.2 (R Core Team, 2019). GENELAND differs from STRUCTURE in that geographical information can be incorporated to produce more accurate inferences of population structure based on the spatial distribution of individuals (Levy et al., 2012).

The spatial structure and posterior probability of belonging to a given cluster were determined using the correlated and null alleles models, for runs of *K* = 1 to *K* = 5, and 10 multiple runs were done to check convergence. Each run consisted of 100,000 MCMC iterations with a thinning of 100 and a burn‐in of 200. The correlated alleles model may be more powerful at detecting subtle differentiation. Thus, the correlated alleles model could be more informative than the default model of uncorrelated frequencies and hence get more accurate results, in particular in presence of low differentiation and when K is unknown (Guillot, 2008). The uncertainty of coordinates was set to 10, as this is a computational step that allows for the inclusion of information on the mobility of the species in question (Guillot et al., 2011), according to the characteristics of the species (Medri et al., 2006) and the landscape (Durigan et al., 2007).

Many types of statistical analyses have been applied to understand how the landscape structures genetic variation in natural populations (Guillot et al., 2005; Manel et al., 2003). Bayesian spatial cluster analysis and edge detection methods are frequently useful for describing patterns and revealing microevolutionary processes between individuals and within populations (Guillot et al., 2009; Safner et al., 2011). Thus, in addition to the Bayesian spatial cluster analysis implemented in GENELAND, we performed the edge detection method in the Alleles in Space (AIS) Software (Miller, 2005).

Monmonier's algorithm (Monmonier, 1973) was implemented in the individual‐based program AIS to detect the presence of barriers to gene flow and evidence of isolation by barrier (IBB). Monmonier's algorithm is supervised, and the number of biogeographical barriers to be calculated must be specified beforehand (Miller, 2005).

We computed the two first barriers that were contiguous to each other, once the forming boundary has closed on itself by forming a loop around a population (Manni et al., 2004). The barriers were inferred using a Delaunay triangulation‐based connectivity network and residual genetic distances (Manni et al., 2004; Monmonier, 1973). This combination of spatial statistical analyses involving Bayesian clustering and barrier detection methods involving Monmonier's algorithm may be a powerful tool for maximizing accuracy and minimizing type‐1 errors in barrier detection (Blair et al., 2012).

Aggregation indices were also calculated in AIS. These indices are usually used in ecological studies to quantify spatial patterns and can also be useful for testing genetic diversity patterns over a given landscape. All of the analyses in AIS were calculated with 100,000 permutations/iterations (Miller, 2005).

To determine whether there was evidence of isolation by distance (IBD), the test provided by Mantel (Mantel, 1967; Sokal, 1979) was performed in the VEGAN package of the R software, version 3.4.2 (Oksanen et al., 2019; R Core Team, 2019) using Spearman's correlation, Euclidean dissimilarity indices for geographic distances, and the Bray–Curtis index for genetic distances. These values were used in the interindividual genetic analyses due to their consistent performance in this context; they effectively replace the genetic distances commonly used in population analyses (Bowlby et al., 2016; Shirk et al., 2017).

Subsequently, we performed the principal component analyses (PCA) of individual genetic variation using R version 4.0.2 (R Core Team, 2019) and ADEGENET 2.1.3, centered and scaled (Jombart, 2008).

The main asset of PCA is the absence of any assumption about the underlying population genetic model, and PCA has been suggested as an alternative to individual‐based analysis such as Bayesian clustering algorithms and edge detection methods (Patterson et al., 2006; Jombart et al., 2009). However, PCA does not provide a group assessment and would require a priori definition of clusters to study population structures (Jombart et al., 2010).

Thus, the subdivision a priori were defined according to the geographic area of the sampled individuals: (a) the population of the conservation area—EENP, (b) the population of the conservation area—EESB, and (c) a population defined by the animals sampled in other areas across the northwest region of São Paulo State—NP. This subdivision allowed us to compare and evaluate the genetic patterns among the animals in conservation areas and animals farther away from the protected areas.

Cluster analysis was also performed in PAST (Hammer et al., 2001) using the same subdivision and Bray–Curtis index (Bowlby et al., 2016; Shirk et al., 2017), according to the Neighbor‐Joining (NJ) method (Saitou and Nei, 1987). The nodes were supported by bootstrap analysis with 10,000 replicates.

Parameters for the evaluation of the microsatellite loci were studied. Estimates of genetic diversity and microsatellite variation in the population of São Paulo State (all sampled individuals) and within the subpopulations of the conservation areas, from which the average allelic richness and gene diversity (HE) were calculated. Finally, genetic divergence between populations was estimated by calculating the unbiased estimate of F_ST_, and the gene flow analysis was computed using the number of migrants per generation based on the method involving private alleles (Barton & Slatkin, 1986; Weir & Cockerham, 1984) using the GENEPOP program, version 4.2 (Raymond & Rousset, 1995).

## 3 RESULTS

### 3.1 Population structure

#### 3.1.1 Bayesian clustering analyses

**Figure 3:**
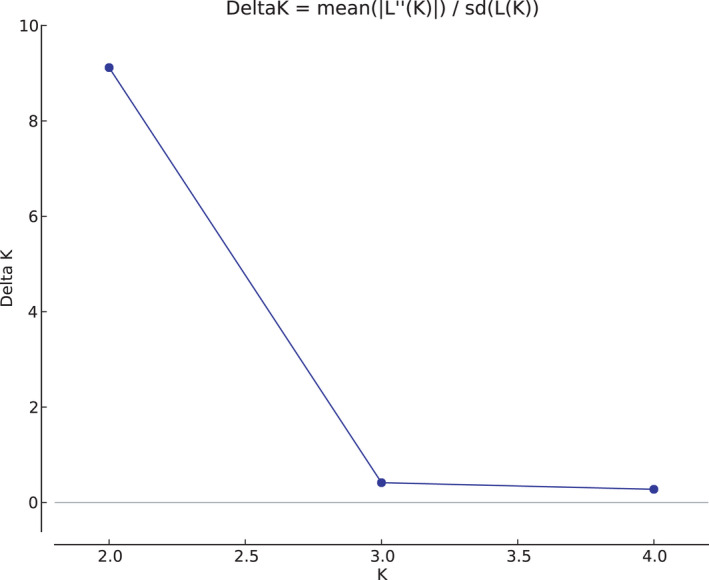
Results of the Structure Harvester showing delta K values from the Evanno method

**Figure 4:**
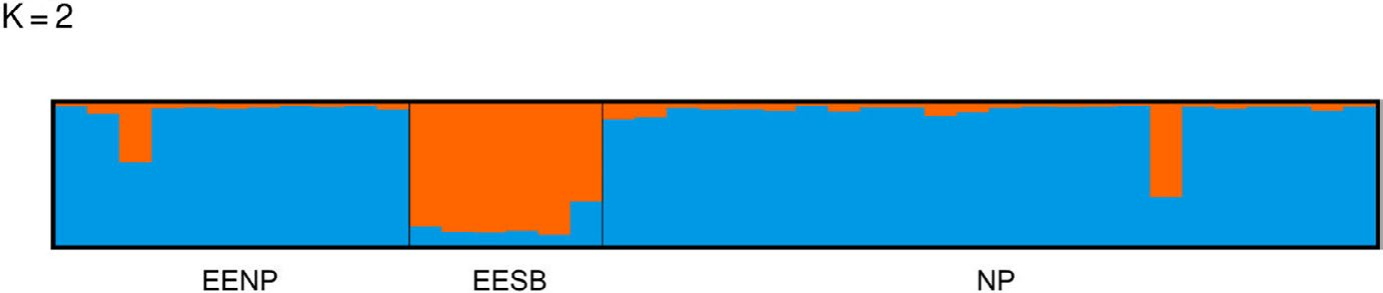
Result of the STRUCTURE Software analysis indicating the presence of two population clusters (*K* = 2) based on the higher Delta K value. Noroeste Paulista Conservation Area population (EENP), Santa Bárbara Conservation Area population (EESB), and isolated individuals sampled in other regions of the northwest area of São Paulo (NP)

**Figure 5:**
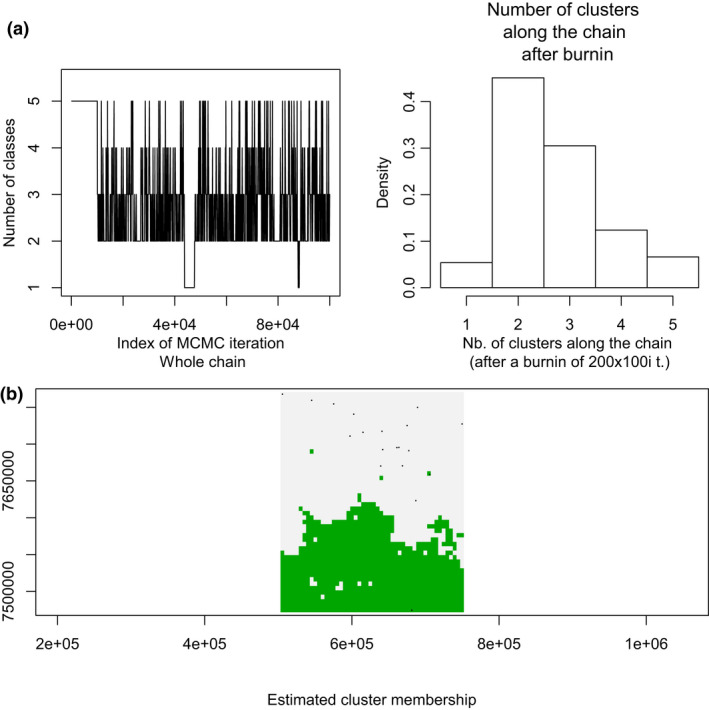
(a) Identification of two clusters using GENELAND (*K* = 2). (b) Map illustrating the structure of the clusters identified. Each color represents a different cluster: cluster 1 (gray), cluster 2 (green)

**Figure 6:**
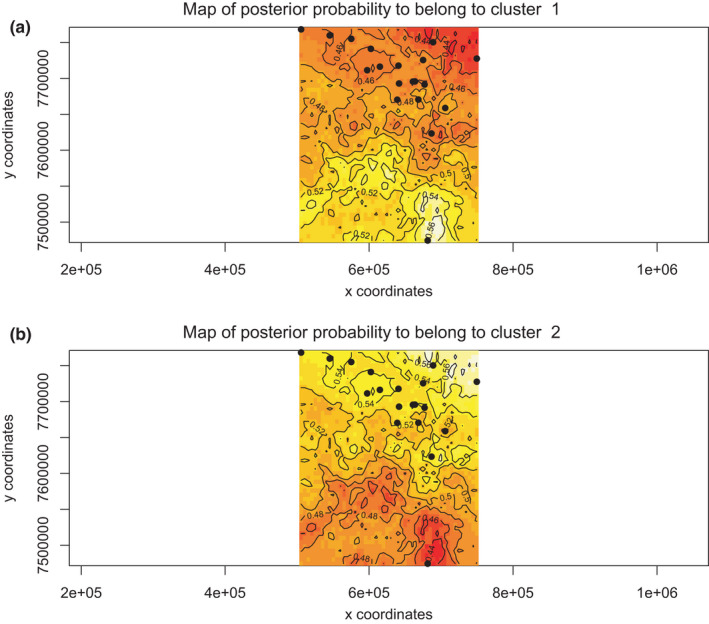
(a) Map showing individual attribution and posterior probability of belonging to the cluster 1. (b) Map showing individual attribution and posterior probability of belonging to the cluster 2. Black dots represent the coordinates of the sites where the animals were obtained. Colors indicate regions of high (lightest yellow) and low (darkest red) posterior probability of belonging to the area or the cluster, and the boundary lines represent spatial changes in the probability of population attribution

The results of all Bayesian clustering methods yielded the same optimal number of clusters. We identified *K* = 2 in STRUCTURE as the most probable number of clusters after calculating ΔK (Figures 3 and 4). These clusters corresponded to the individuals of the northwestern region of São Paulo State (blue) and central‐southern region/ EESB individuals (orange). In the spatially explicit analyses performed in GENELAND, the most probable number of clusters also was *K* = 2 (Figure 5) and the results showed that the individuals are spatially structured across the area (F_ST_ = 0.03407 in the dataset containing all individuals and F_ST_ = 0.09 in the dataset containing only the populations of conservation areas). The posterior probability maps indicated that the individuals belong to specific clusters, with a low probability of occupation in farther away areas (Figure 6).

Despite the low Delta *K* value (9.118790) and the posterior probability for individuals belonging to cluster 1 and cluster 2 (<0.56), both results indicate the population subdivision and corroborate the hypothesis that there are two groups in the studied area. Besides, the relatively low values may be due to the experimental sampling method and the number of individuals used.

#### 3.1.2 Isolation by barrier and Isolation by distance

Monmonier's algorithm placed the main boundary separating the individuals/populations of the northwestern region and the central‐southern region. The other contiguous barrier showed that the individuals sampled in the most urbanized area are isolated (including the EENP area population), and the result is suggestive of isolation by barrier—IBB (figure not shown).

The Mantel test was performed to test the isolation by distance (IBD) hypothesis, and no evidence of IBD was found (figure not shown) since there was no correlation between genetic and geographic distances (*r* = 0.06982, *p* = .195). The allelic aggregation index, which is calculated based on the overall spatial distribution of all of the alleles and loci (RjAVE), was 0.778477556 (*p* = .00633). This result indicates that the samples presented a clumped spatial distribution (RjAVE < 1) and supports the structure found in the Bayesian spatial analysis.

In other words, the analyses as a whole indicate that spatial structuring does exist, but that distance is not an isolated factor that led to this population structure pattern.

### 3.2 Multivariate analysis

#### 3.2.1 Principal component analysis

**Figure 7:**
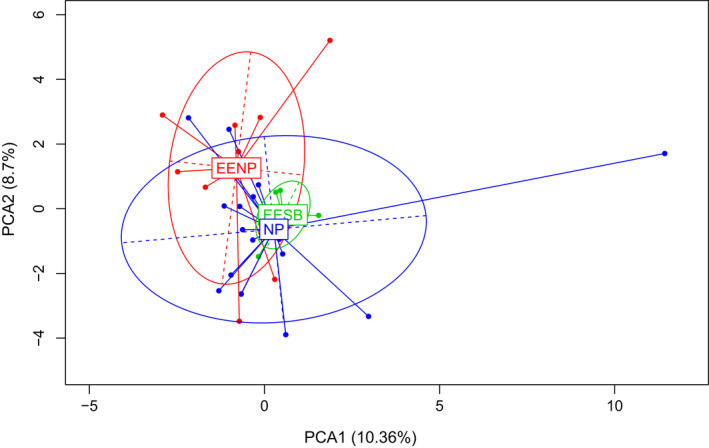
PCA result based on the alleles and geographic position of the individuals/populations: Noroeste Paulista Conservation Area population (EENP), Santa Bárbara Conservation Area population (EESB), and isolated individuals sampled in other regions of the northwest area of São Paulo (NP)

The principal component analysis (PCA) was carried out to define in few vectors all data collected from the genotypes studied (Figure 7). The PCA results indicated that only 5 components explain the variation of the data set. The numbers of principal components have been defined according to the relationship between the eigenvalues. The first component (PC1, eigenvalue = 5.283) contributed by 10.358% of the total variability, while PC2 (eigenvalue = 4.435) accounted for 8.696% of the total variability. The distribution of genotypes in the PCA analysis revealed the differences in the distribution of the allelic frequencies in the conservation area populations.

EENP population diverges from the others (NP and EESB). Also, NP and EESB have the same average, but NP has much greater variation than EESB. This variation was expected due to the number of individuals sampled per population. It is important to emphasize that the existing variability in the total population of the state of São Paulo shows the importance of the conservation of these populations for the maintenance of the genetic resources to the species *M. tridactyla*.

The EESB population showed less variation, but a similar average to the NP population (ellipses centers almost together), and the EENP population stands out mainly to PC2. Each ellipse comprises (in general) 95% of the population, and this explains the elements that are external to the ellipse (outliers).

#### 3.2.2 Clustering methods

The dendrogram based on the NJ algorithm revealed two discrete clusters.

**Figure 8:**
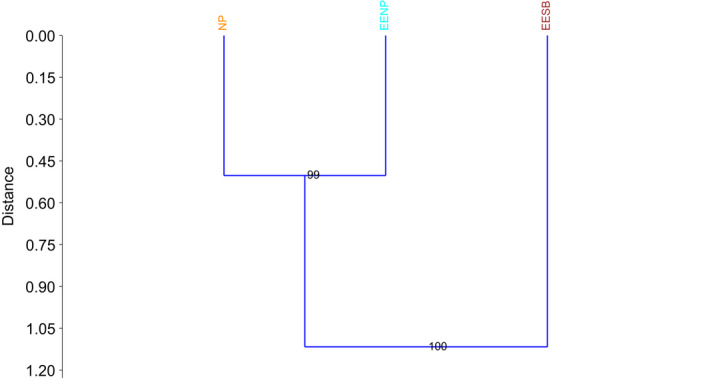
Dendrogram (NJ) showing the genetic distance of the populations: Noroeste Paulista Conservation Area population (EENP), Santa Bárbara Conservation Area population (EESB), and isolated individuals sampled in other regions of the northwest area of São Paulo (NP)

Results presented high bootstrap support values (Figure 8), a finding that corroborates the results of the individual‐based clustering analyses.

### 3.3 Genetic diversity and population differentiation

The population of São Paulo State (all sampled individuals) presented high genetic diversity, both in relation to the expected heterozygosity (0.7188) and in relation to allelic richness (total number of alleles = 51; mean number of alleles = 8.5 alleles/loci).

Descriptive analyses indicate that all of the loci were highly polymorphic. After the application of Bonferroni's correction, two loci exhibited deviations from Hardy–Weinberg equilibrium, and the tests for linkage disequilibrium were found not to be statistically significant (*p* > .05). Analyses were also performed between individuals found close to the EENP conservation area (*n* = 10) and those found close to the EESB conservation area (*n* = 6) to investigate the genetic diversity and differentiation between the population of the conservation areas. The results reflected greater genetic differentiation between these two groups (F_ST_ = 0.1739) and relatively low/moderate F_IT_ (0.1348) and F_IS_ (0.0912) values.

### 3.4 Gene flow analysis

Gene flow analyses were carried out for testing levels of gene flow among individuals belonging to different areas, and the results showed that the number of migrants per generation is lower between two areas that are farther away from each other. This reduction in the migration rate at regional levels may have led to population differentiation. Also, we were able to infer migration between the animals that were obtained in the portions of the landscape surrounding the conservation areas and the result indicated a lower level of migration between the populations of the EENP and EESB (N_m_ = 0.615729).

## 4 DISCUSSION

### 4.1 Genetic diversity of *Myrmecophaga tridactyla*

The use of microsatellite markers in this study aided in the identification of substantial intrapopulation variance, population subdivision (*K* = 2), and high rates of genetic diversity. The genetic structure of the giant anteater populations in this region is best explained by the presence of barriers to gene flow than by geographic distance.

These discoveries, supported by different statistical methods, highlight the importance of small‐scale genetic studies to determine whether any barriers are restricting gene flow and, ultimately, to prevent further losses of genetic diversity. Identifying population numbers, levels of genetic diversity, and the degree of connectivity between individuals and populations in an anthropized landscape may provide useful information for conservation strategies and demonstrated the importance of preserving the few habitat fragments that remain (Sullivan et al., 2019; Weiss & Leese, 2016).

Few studies have been performed to evaluate population dynamics and the genetic variability of giant anteater populations (Clozato et al., 2017; Collevatti et al., 2007). Garcia et al. (2005) developed six species‐specific microsatellite primers and evaluated allelic diversity of markers from fifteen individuals (eight animals that had been run over in the states of São Paulo and Mato Grosso, and seven that had been collected in the state of Goiás). They obtained levels of genetic diversity similar to those found in our study (*H*_o_ = 0.61, *H*_e_ = 0.63, and 26 alleles total), considering the small sample size.

Using the same microsatellite markers, Collevatti et al. (2007) analyzed 27 giant anteaters from Emas National Park (which is also located across the states of São Paulo and Mato Grosso) and found low levels of genetic diversity (mean number of alleles = 3; *H*_e_ = 0.482 and *H*_o_ = 0.059), as well as a high degree of inbreeding (*F*_IT_ = 0.879). According to the authors, the low level of diversity in these loci may be the result of the natural history and evolution of *M. tridactyla*, or it may be a consequence of population bottlenecks due to the decades‐long history of recurrent fires in the area (Collevatti et al., 2007; Silveira et al., 1999).

The high rates of genetic diversity found were unexpected: The populations are structured, exhibit genetic differentiation, and are located in a fragmented habitat, factors which can decrease genetic diversity (Frankham, 2005). However, this relationship is not always linear; thus, not all large populations will exhibit high genetic diversity, and not all bottlenecked or decimated populations will experience a reduction in genetic diversity (Torres‐Florez et al., 2014). Furthermore, changes to genetic diversity associated with habitat fragmentation may be subtle due to recent environmental disturbances (Dixo et al., 2009).

Though some species are highly sensitive to human activity and experience local extinction and/or population reduction when urban development begins, other species are able to persevere as small and relatively isolated populations (Ramalho et al., 2018). As a consequence, in a short period and without the occurrence of extinction, many populations are expected to exhibit fixation more rapidly, but to retain greater overall genetic diversity than a single large population. However, in the long term, when small populations become extinct, the remaining small populations will retain less genetic diversity than the original single large population (Frankham et al., 2002). Therefore, the genetic consequences of habitat fragmentation in a single large population are different from the genetic consequences for various small and isolated population fragments, even in large protected areas.

The genetic patterns observed in this study may be explained by environmental, evolutionary, and biological factors that have led to regional differences in the genetic frequency of isolated populations. Levels of genetic diversity may be maintained or lost through various types of selection, as well as by the effects of genetic drift in selectively neutral mutations in finite populations (Charlesworth et al., 1997; Crow & Kimura, 1970; Gillespie, 1991; Kimura, 1983).

### 4.2 Barriers to gene flow

Barriers to gene flow were identified, and it can be inferred that the barriers have reduced connectivity between giant anteater populations and a lack of migrations between fragments located farther away from each other may lead to population differentiation (and likely between populations of many other land species).

The results of Bayesian analyzes, edge detection method, and PCA were slightly different. Nevertheless, these results showed the separation of the EENP individuals and EESB individuals. Also, the number of migrants per generation calculated for the populations of the conservation areas (N_m_ = 0.615729) indicates the subdivision of the population since rates lower than one migrant per generation are insufficient for counteracting the effects of genetic drift (Slatkin, 1993). Inferring about the cause of the barrier was not possible due to the characteristics of species, population dynamics, and even the fragmentation period in the area, that make it difficult to distinguish between recent and historical signatures of interrupted gene flow in our experimental scheme (Dixo et al., 2009). The main discrepancy found in the analyses was the results of the PCA and the other analyses such as NJ and Bayesian methods, that differently grouped the individuals sampled throughout the northwest of São Paulo (NP). This difference may be the result of the experimental sampling used in the present study and must be carefully considered in the identification of clusters when the genetic difference is low. On the other hand, the methods presented here proved to be effective in the differentiation of clusters in populations with moderate/high genetic differences, as in the case of the EESB and EENP individuals, even with few individuals sampled per population.

In general, the current results show that barriers have prevented gene flow at the regional level, but migrations may have occurred at shorter distances and may have increased intrapopulation variation at the local level contributing to the sustained genetic diversity observed herein (Barton, 2008). Following this logic, differences between the most geographically distant populations of São Paulo State would have increased over time and led to cluster differentiation, but with substantial genetic variation at short distances due to probable short‐distance migrations.

Population subdivision may also lead to a geographic structure that affects allele frequencies in space and proportions of different genotypes in local populations (Garnier‐Géré & Chikhi, 2013). Thus, gene flow does not depend on distance alone, but also on the nature of the landscape surrounding and between the populations; the separation of populations may have favored the retention of genetic variants in small and isolated populations (Templeton et al., 2001).

### 4.3 Biological characteristics of the species

In addition to evolutionary and environmental factors, certain species’ biological characteristics may influence the degree of genetic diversity loss in populations; for example, a long life span seems to contribute to sustained genetic variability in wild populations (Torres‐Florez et al., 2014). The processes that lead to the loss of genetic diversity may be buffered by intrinsic biological characteristics, and long generation times may result in small populations that seem genetically diverse despite periods of substantial decline (Hailer et al., 2006).

The estimated lifespan of the giant anteater is more than 15 years in the wild and 20–30 years in captivity. The species’ generation time is estimated to be approximately seven years (Gaudin et al., 2018; Knott et al., 2013; Medri et al., 2006; Miranda et al., 2014; Nowak, 2018). Studies on long‐living mammals have identified high rates of genetic variability in many small and isolated populations; in some cases, even species that are almost extinct still exhibit high genetic diversity (Dinerstein & Mccracken, 1990; Swart et al., 1994; Taylor et al., 1994; Zavala‐Páramo et al., 2017).

Species with shorter generation times and smaller body sizes typically tend to experience faster genetic responses to man‐made barriers (Epps et al., 2005). Therefore, a loss in genetic diversity in a long‐living species cannot be easily detected in a short period of time, and this factor should be considered when evaluating the species’ risk of extinction. Thus, it is plausible that the long life span of the giant anteater combined with its demographic and evolutionary history delayed the loss of the genetic diversity in these populations.

Other hypotheses to explain the processes that have maintained genetic diversity in these populations should not be discarded, but regardless of the hypotheses and theories that explain population patterns, the analyses performed herein revealed that giant anteaters in the state of São Paulo should be a priority in conservation measures due to their high genetic diversity and variability.

### 4.4 Conservation issues

Genetic criteria and allelic richness in particular are crucial tools for selecting candidate populations of wild species to be prioritized for conservation (Petit et al., 2008). Thus, despite this persistently high genetic variability, factors such as habitat fragmentation, habitat destruction, and demographic instability may lead wild populations to extinction before the genetic response to environmental impact becomes clear, particularly in long‐living species (Lacy, 1997).

Conservation plans are therefore essential to increase the chances of giant anteater survival and to enable the conservation of genetic resources for subsequent species recovery (Théry, 2011). Due to the use of convenience sampling in this study, the degree of genetic diversity observed may be the result of the use of various study sites with one or more individuals; but in any case, these individuals as a whole represent substantial genetic richness for a species living in an anthropized region. The findings reported herein indicate that management plans must be implemented at local and regional levels because, in the context of intense human activity, the giant anteater population is likely to be limited to a few individuals isolated in the remaining suitable habitats around the state (Bertassoni et al., 2019).

The frequency at which giant anteaters are run over on local highways shows that road ecology studies are necessary to determine local mitigating measures to consider when implementing fauna passage systems; the ultimate goal of this research and these policy measures should be to prevent the decline of animal populations, including that of the giant anteater (Teixeira et al., 2017).

Finally, conservation genetic studies on small geographic scales may provide important information for determining the status of threatened species populations. It is also crucial that interactions between scientists and policymakers improve so that the results of genetic and ecological research can be applied in the establishment of effective conservation strategies (Montgelard et al., 2014; Vernesi et al., 2008).

## 5 FINAL CONSIDERATIONS

This study has shown that population of *M. tridactyla* in the state of São Paulo, including the conservation areas, maintain high levels of genetic diversity. Though the sample size is relatively small, it seems to be the largest sample size in conservation genetics studies performed on the giant anteater at the regional level available in the literature.

The results emphasize the importance of fragmented areas for the maintenance of genetic diversity in anthropogenically modified landscapes and support the creation of adequate conservation plans that can work to prevent the local extinction of genetically important populations.

Environmental causes such as roadkills and the increasing agricultural borders can lead populations to local extinctions before a perceptible genetic response to contemporary habitat fragmentation. Thus, the populations in the state of São Paulo should take priority in the conservation of the genetic resources of the species.

## CONFLICT OF INTEREST

None declared.

## AUTHOR CONTRIBUTION

**Ricardo Sartori:** Data curation (equal); Formal analysis (equal); Investigation (lead); Methodology (lead); Project administration (equal); Resources (equal); Software (lead); Visualization (equal); Writing‐original draft (supporting); Writing‐review & editing (lead). **Alessandro Garcia Lopes:** Methodology (equal); Visualization (equal); Writing‐review & editing (equal). **Luiz Paulo Nogueira Aires:** Methodology (equal); Visualization (equal); Writing‐review & editing (equal). **Rita de Cassia Bianchi:** Formal analysis (equal); Funding acquisition (equal); Validation (equal); Visualization (equal); Writing‐review & editing (equal). **Cinara Cássia Brandão:** Formal analysis (equal); Supervision (supporting); Validation (equal); Visualization (equal); Writing‐review & editing (equal). **Adriana Coletto Morales:** Formal analysis (equal); Validation (equal); Visualization (equal); Writing‐review & editing (equal). **Lilian Castiglioni:** Conceptualization (lead); Data curation (equal); Formal analysis (equal); Funding acquisition (lead); Project administration (lead); Resources (equal); Supervision (lead); Validation (lead); Visualization (equal); Writing‐original draft (lead); Writing‐review & editing (equal).

## Data Availability

Sampling locations and microsatellite genotypes are archived in the Dryad Data Repository (https://doi.org/10.5061/dryad.cfxpnvx31).

## References

[ece36809-bib-0001] Arif, I. A. , Khan, H. A. , Shobrak, M. , Al Homaidan, A. A. , Al Sadoon, M. , Al Farhan, A. H. , & Bahkali, A. H. (2010). Interpretation of electrophoretograms of seven microsatellite loci to determine the genetic diversity of the Arabian Oryx. Genetics and Molecular Research, 9, 259–265. 10.4238/vol9-1gmr714 20198581

[ece36809-bib-0002] Barton, N. H. (2008). The effect of a barrier to gene flow on patterns of geographic variation. Genetics Research, 90(1), 139–149. 10.1017/S0016672307009081 18289408

[ece36809-bib-0003] Barton, N. H. , & Slatkin, M. A. (1986). Quasi‐equilibrium theory of the distribution of rare alleles in a subdivided population. Heredity, 56, 409–415. 3733460 10.1038/hdy.1986.63

[ece36809-bib-0004] Bertassoni, A. , Costa, R. T. , Gouvea, J. A. , Bianchi, R. D. C. , Ribeiro, J. W. , Vancine, M. H. , & Ribeiro, M. C. (2019). Land‐use changes and the expansion of biofuel crops threaten the giant anteater in southeastern Brazil. Journal of Mammalogy, 100(2), 435–444. 10.1093/jmammal/gyz042

[ece36809-bib-0005] Blair, C. , Weigel, D. E. , Balazik, M. , Keeley, A. T. H. , Walker, F. M. , Landguth, E. , Cushman, S. , Murphy, M. , Waits, L. , & Balkenhol, N. (2012). A simulation‐based evaluation of methods for inferring linear barriers to gene flow. Molecular Ecology Resources, 12(5), 822–833. 10.1111/j.1755-0998.2012.03151.x 22551194

[ece36809-bib-0006] Bowlby, H. D. , Fleming, I. A. , & Gibson, A. J. F. (2016). Conservation Genetics, 17, 823.

[ece36809-bib-0007] Butchart, S. H. M. , Walpole, M. , Collen, B. , Strien, A. , Scharlemann, J. P. W. , Almond, R. E. A. , Baillie, J. E. M. , Bomhard, B. , Brown, C. , Bruno, J. , Carpenter, K. E. , Carr, G. M. , Chanson, J. , Chenery, A. M. , Csirke, J. , Davidson, N. C. , Dentener, F. , Foster, M. , Galli, A. , … Watson, R. (2010). Global biodiversity: Indicators of recent declines. Science, 328, 1164. 20430971 10.1126/science.1187512

[ece36809-bib-0008] Canale, G. R. , Peres, C. A. , Guidorizzi, C. E. , Gatto, C. A. F. , & Kierulff, M. C. M. (2012). Pervasive defaunation of forest remnants in a tropical biodiversity hotspot. PLoS One, 7(8). 10.1371/journal.pone.0041671 PMC3419225 22905103

[ece36809-bib-0009] Carvalho, F. M. V. , De Marco, P. , & Ferreira, L. G. (2009). The Cerrado into‐pieces: Habitat fragmentation as a function of landscape use in the savannas of central Brazil. Biol. Conser, 142, 1392–1403. 10.1016/j.biocon.2009.01.031

[ece36809-bib-0010] Charlesworth, B. , Nordborg, M. , & Charlesworth, D. (1997). The effects of local selection, balanced polymorphism and background selection on equilibrium patterns of genetic diversity in subdivided populations. Genetical Research, 70(2), 155–174. 9449192 10.1017/s0016672397002954

[ece36809-bib-0012] Clozato, C. L. , Miranda, F. R. , Lara‐Ruiz, P. , Collevatti, R. G. , & Santos, F. R. (2017). Population structure and genetic diversity of the giant anteater (*Myrmecophaga tridactyla:* Myrmecophagidae, Pilosa) in Brazil. Genetics and Molecular Biology, 40, 1 10.1590/1678-4685-gmb-2016-0104 28199447 PMC5409771

[ece36809-bib-0013] Collevatti, R. G. , Leite, K. C. E. , Miranda, G. H. B. , & Rodrigues, F. H. G. (2007). Evidence of high inbreeding in a population of the endangered giant anteater, *Myrmecophaga tridactyla* (Myrmecophagidae), from Emas National Park. Brazil. Genetics and Molecular Biology, 30(1), 112–120. 10.1590/S1415-47572007000100020

[ece36809-bib-0014] Crow, J. F. , & Kimura, M. (1970). An introduction to population genetics theory. Harper and Row.

[ece36809-bib-0015] Dempster, A. P. , Laird, N. M. , & Rubin, D. B. (1977). Maximum likelihood from incomplete data via the EM algorithm. Journal of the Royal Statistical Society. Series B, 39(1), 1–38.

[ece36809-bib-0016] Dinerstein, E. , & McCracken, G. F. (1990). Endangered greater one‐horned rhinoceros carry high levels of genetic variation. Conservation Biology, 4(4), 417–422. 10.1111/j.1523-1739.1990.tb00316.x

[ece36809-bib-0017] Diniz, M. F. , & Brito, D. (2013). Threats to giant anteater, *Myrmecophaga tridactyla* (Pilosa: Myrmecophagidae), viability in a protected Cerrado remnant encroached by urban expansion in central Brazil. Zoologia, 30, 151–156.

[ece36809-bib-0018] Diniz, M. F. , & Brito, D. (2015). Protected areas effectiveness in maintaining viable giant anteater (*Myrmecophaga tridactyla*) populations in an agricultural frontier. Revista Natureza E Conservação, 13(2), 145–151. 10.1016/j.ncon.2015.08.001

[ece36809-bib-0019] Dixo, M. , Metzger, J. P. , Morgante, J. S. , & Zamudio, K. R. (2009). Habitat fragmentation reduces genetic diversity and connectivity among toad populations in the Brazilian Atlantic Coastal Forest. Biological Conservation, 142(8), 1560–1569. 10.1016/j.biocon.2008.11.016

[ece36809-bib-0020] Don, R. , Cox, P. , Wainwright, B. , Baker, K. , & Mattick, J. S. (1991). Touchdown PCR to circumvent spurious priming during gene amplification. Nucleic Acids Research, 19(14), 4008 10.1093/nar/19.14.4008 1861999 PMC328507

[ece36809-bib-0021] Durigan, G. , Siqueira, M. F. , & Franco, G. A. D. C. (2007). Threats to the Cerrado remnants of the state of São Paulo, Brazil. Scientia Agricola, Piracicaba, Brazil, 64(4), 355–363. 10.1590/S0103-90162007000400006

[ece36809-bib-0022] Earl, D. A. , & Vonholdt, B. M. (2012). Structure Harvester: A website and program for visualizing STRUCTURE output and implementing the Evanno method. Conservation Genetics Resources, 4, 359–361. 10.1007/s12686-011-9548-7

[ece36809-bib-0023] Epps, C. W. , Palsboll, P. J. , Wehausen, J. D. , Roderick, G. K. , Ramey, R. R. , & McCullough, D. R. (2005). Highways block gene flow and cause a rapid decline in genetic diversity of desert bighorn shee. Ecology Letters, 8(10), 1029–1038.

[ece36809-bib-0024] Evanno, G. , Regnaut, S. , & Goudet, J. (2005). (2005) Detecting the number of clusters of individuals using the software STRUCTURE: A simulation study. Molecular Ecology, 14, 2611–2620. 10.1111/j.1365-294X.2005.02553.x 15969739

[ece36809-bib-0025] Fahrig, L. (2003). Effects of habitat fragmentation on biodiversity. Annual Review of Ecology, Evolution, and Systematics, 34, 487–515.

[ece36809-bib-0026] Falush, D. , Stephens, M. , & Pritchard, J. K. (2003). Inference of population structure using multilocus genotype data: Linked loci and correlated allele frequencies. Genetics, 164(4), 1567–1587. 12930761 10.1093/genetics/164.4.1567 PMC1462648

[ece36809-bib-0027] Frankham, R. (2005). Genetics and extinction. Biological Conservation, 126, 131–140. 10.1016/j.biocon.2005.05.002

[ece36809-bib-0028] Frankham, R. , Ballou, J. D. , & Briscoe, D. A. (2002). Introduction to conservation genetics. Cambridge University Press.

[ece36809-bib-0029] Garcia, J. E. , Vilas Boas, L. A. , Lemos, M. V. F. , de Macedo Lemos, E. G. , & Contel, E. P. B. (2005). Identification of microsatellite DNA markers for the giant anteater *Myrmecophaga tridactyla* . Journal of Heredity, 96, 600–602. 10.1093/jhered/esi089 15994414

[ece36809-bib-0030] Garnier‐Géré, P. , & Chikhi, L. (2013). Population subdivision, Hardy–Weinberg equilibrium and the wahlund effect In: Encyclopedia of Life Sciences (eLS). Hoboken: John Wiley & Sons, Ltd (Ed.) 10.1002/9780470015902.a0005446.pub3

[ece36809-bib-0031] Gaudin, T. J. , Hicks, P. , & di Blanco, Y. (2018). *Myrmecophaga tridactyla* (Pilosa: Myrmecophagidae). Mammalian Species, 50(956), 1–13. 10.1093/mspecies/sey001

[ece36809-bib-0032] Gillespie, J. H. (1991). The causes of molecular evolution. Oxford University Press.

[ece36809-bib-0033] Guillot, G. (2008). Inference of structure in subdivided populations at low levels of genetic differentiation‐the correlated allele frequencies model revisited. Bioinformatics, 24(19), 2222–2228. 10.1093/bioinformatics/btn419 18710873

[ece36809-bib-0034] Guillot, G. , Leblois, R. , Coulon, A. , & Frantz, A. C. (2009). Statistical methods in spatial genetics. Molecular Ecology, 18, 4734–4756. 19878454 10.1111/j.1365-294X.2009.04410.x

[ece36809-bib-0035] Guillot, G. , Mortier, F. , & Estoup, A. (2005). GENELAND: A computer package for landscape genetics. Molecular Ecology Notes, 5, 712–715. 10.1111/j.1471-8286.2005.01031.x

[ece36809-bib-0036] Guillot, G. , Santos, F. , & Estoup, A. (2011) Population genetics analysis using R and the Geneland program. https://orbit.dtu.dk/en/publications/population‐genetics‐analysis‐using‐r‐and‐the‐geneland‐program

[ece36809-bib-0037] Hailer, F. , Helander, B. , Folkestad, A. O. , Ganusevich, S. A. , Garstad, S. , Hauff, P. , Koren, C. , Nygård, T. , Volke, V. , Vilà, C. , & Ellegren, H. (2006). Bottlenecked but long‐lived: High genetic diversity retained in white‐tailed eagles upon recovery from population decline. Biology Letters, 22(2), 316–319. 10.1098/rsbl.2006.0453 PMC1618921 17148392

[ece36809-bib-0038] Hammer, Ø. , Harper, D. A. T. , & Ryan, P. D. (2001). PAST: Paleontological statistics software package for education and data analysis. Palaeontologia Electronica, 4(1), 9.

[ece36809-bib-0039] Hijmans, R. J. , Guarino, L. , Bussink, C. , Mathur, P. , Cruz, M. , Barrentes, I. , Rojas, E. (2012) DIVA‐GIS 7.5. A geographic information system for the analysis of species distribution data. http://www.diva‐gis.or

[ece36809-bib-0040] IF – INSTITUTO FLORESTAL (2011). Plano de Manejo da Estação Ecológica de Santa Bárbara. Governo do Estado de São Paulo.

[ece36809-bib-0041] IF – INSTITUTO FLORESTAL . (2014). Proposta de criação da floresta estadual do Noroeste Paulista. Governo do Estado de São Paulo.

[ece36809-bib-0042] Jombart, T. (2008). adegenet: A R package for the multivariate analysis of genetic markers. Bioinformatics, 24(11), 1403–1405. 10.1093/bioinformatics/btn129 18397895

[ece36809-bib-0043] Jombart, T. , Devillard, S. , & Balloux, F. (2010). Discriminant analysis of principal components: A new method for the analysis of genetically structured populations. BMC Genetics, 11, 94 10.1186/1471-2156-11-94 20950446 PMC2973851

[ece36809-bib-0044] Jombart, T. , Pontier, D. , & Dufour, A. B. (2009). Genetic markers in the playground of multivariate analysis. Heredity (Edinb), 102(4), 330–341. 10.1038/hdy.2008.130 19156164

[ece36809-bib-0045] Keinath, D. A. , Doak, D. F. , Hodges, K. E. , Prugh, L. R. , Fagan, W. , Sekercioglu, C. H. , Buchart, S. H. M. , & Kauffman, M. (2017). A global analysis of traits predicting species sensitivity to habitat fragmentation. Global Ecology and Biogeography, 26, 115–127. 10.1111/geb.12509

[ece36809-bib-0046] Kimura, M. (1983). The neutral theory of molecular evolution. Cambridge University Press.

[ece36809-bib-0047] Knott, K. K. , Roberts, B. M. , Maly, M. A. , Vance, C. K. , DeBeachaump, J. , Majors, J. , Riger, P. , DeCaluwe, H. , & Kouba, A. J. (2013). Fecal estrogen, progestagen, and glucocorticoid metabolites during the estrous cycle and pregnancy in the giant anteater (*Myrmecophaga tridactyla*): Evidence for delayed implantation. Reproductive Biology and Endocrinology, 11, 83 10.1186/1477-7827-11-83 23981950 PMC3765926

[ece36809-bib-0048] Kopelman, N. M. , Mayzel, J. , Jakobsson, M. , Rosenberg, N. A. , & Mayrose, I. (2015). CLUMPAK: A program for identifying clustering modes and packaging population structure inferences across K. Molecular Ecology Resources, 15(5), 1179–1191. 25684545 10.1111/1755-0998.12387 PMC4534335

[ece36809-bib-0049] Korbie, D. J. , & Mattick, J. S. (2008). TouchDown PCR for increased specificity and sensitivity in PCR amplification. Nature Protocols, 3, 1452–1456. 18772872 10.1038/nprot.2008.133

[ece36809-bib-0050] Kronka, F. J. N. , Nalon, M. A. , Matsukuma, C. K. , Kanashiro, M. M. , Shin‐Ike, M. S. , Pavão, M. , Durigan, G. , Lima, L. M. P. R. , Guillaumon, J. R. , Baitello, J. B. , Borgo, S. C. , Manetti, L. A. , Barradas, A. M. F. , Fukuda, J. C. , Shida, C. N. , Barbosa, O. , Soares, A. P. (2005). Inventário florestal da vegetação natural do estado de São Paulo (200 p). Secretaria do Meio Ambiente; Instituto Florestal; Imprensa Oficial.

[ece36809-bib-0051] Lacy, R. C. (1997). Importance of genetic variation to the viability of mammalian populations. Journal of Mammalogy, 78(2), 320–335. 10.2307/1382885

[ece36809-bib-0052] Landguth, E. L. , Fedy, B. C. , OYLER‐McCANCE, S. J. , Garey, A. L. , Emel, S. L. , Mumma, M. , Wagner, H. H. , Fortin, M.‐J. , & Cushman, S. A. (2012). Effects of sample size, number of markers, and allelic richness on the detection of spatial genetic pattern. Molecular Ecology Resources, 12, 276–284. 10.1111/j.1755-0998.2011.03077.x

[ece36809-bib-0053] Levy, E. , Kennington, W. , Tomkins, J. , & Lebas, N. (2012). Phylogeography and population genetic structure of the ornate dragon lizard, *Ctenophorus ornatus* . PLoS One, 7, e46351 10.1371/journal.pone.0046351 23049697 PMC3462208

[ece36809-bib-0054] Manel, S. , Schwartz, M. K. , Luikart, G. , & Taberlet, P. (2003). Landscape genetics: Combining landscape ecology and population genetics. Trends in Ecology and Evolution, 18(4). 10.1016/S0169-5347(03)00008-9

[ece36809-bib-0055] Mank, J. , & Avise, J. (2004). Individual organisms as units of analysis: Bayesian‐clustering alternatives in population genetics. Genetical Research, 84(43). 10.1017/S0016672304007190 15822602

[ece36809-bib-0056] Manni, F. , Guerard, E. , & Heyer, E. (2004) Geographic patterns of (genetic, morphologic, linguistic) variation: how barriers can be detected by using Monmonier's algorithm. Human Biology, 76(2), 173–190. 10.1353/hub.2004.0034 15359530

[ece36809-bib-0057] Mantel, N. (1967). The detection of disease clustering and a generalized regression approach. Cancer Research, Birmingham, 27(2), 209–220. 6018555

[ece36809-bib-0058] Medri, I. M. , Mourão, G. , Rodriguez, F. (2006). Ordem Xenarthra In Reis N. R., Perachi A. L., Pedro W. A., and Lima I. P., (Eds.), Mamíferos do Brasil (pp. 71–99). Universidade Estadual de Londrina.

[ece36809-bib-0059] Miller, M. P. (2005). Alleles in space (AIS): Computer software for the joint analysis of interindividual spatial and genetic information. Journal of Heredity, 96(6), 722–724. 10.1093/jhered/esi119 16251514

[ece36809-bib-0060] Miranda, F. , Bertassoni, A. , & Abba, A. M. (2014) Myrmecophaga tridactyla. The IUCN Red List of Threatened Species.

[ece36809-bib-0061] Miranda, F. R. , Chiarello, A. G. , Röhe, F. et al (2015). Avaliação do Risco de Extinção de *Myrmecophaga tridactyla* Linnaeus, 1758 no Brasil. Processo de avaliação do risco de extinção da fauna brasileira http://www.icmbio.gov.br/portal/biodiversidade/fauna‐brasileira/lista‐de‐especies/7049‐mamiferos‐myrmecophaga‐tridactyla‐tamandua‐bandeira.html

[ece36809-bib-0062] Monmonier, M. S. (1973). Maximum‐difference barriers: An alternative numerical regionalization method. Geographical Analysis, 3, 245–261. 10.1111/j.1538-4632.1973.tb01011.x

[ece36809-bib-0063] Montgelard, C. , Zenboudji, S. , Ferchaud, A.‐L. , Arnal, V. , & van Vuuren, B. J. (2014). Landscape genetics in mammals. Mammalia, 78(2). 10.1515/mammalia-2012-0142

[ece36809-bib-0064] Morris, W. F. , Pfister, C. A. , Tuljapurkar, S. , Haridas, C. V. , Boggs, C. L. , Boyce, M. S. , Bruna, E. M. , Church, D. R. , Coulson, T. , Doak, D. F. , Forsyth, S. , Gaillard, J.‐M. , Horvitz, C. C. , Kalisz, S. , Kendall, B. E. , Knight, T. M. , Lee, C. T. , & Menges, E. S. (2008). Longevity can buffer plant and animal populations against changing climatic variability. Ecology, 89, 19–25. 10.1890/07-0774.1 18376542

[ece36809-bib-0065] Myers, N. , Mittermeier, R. A. , Mittermeier, C. G. , da Fonseca, G. A. B. , & Kent, J. (2000). Biodiversity hotspots for conservation priorities. Nature, 403, 853–858. 10.1038/35002501 10706275

[ece36809-bib-0066] Nowak, R. M. (2018). Walker's Mammals of the World: Monotremes, Marsupials, Afrotherians and Sundatherians, 7th ed Johns Hopkins University Press.

[ece36809-bib-0067] Oksanen, J. , Blanchet, F. G. , Michael, F. , Kindt, R. , Legendre, P. , Mcglinn, D. , Minchin, P. R. , O'hara, R. B. , Simpson, G. L. , Solymos, P. , Stevens, M. H. H. , Szoecs, E. , Wagner, H. (2019). vegan: Community Ecology Package. R package version 2.5.4.

[ece36809-bib-0068] Patterson, N. , Price, A. L. , & Reich, D. (2006). Population structure and eigenanalysis. PLoS Genetics, 2, 2074–2093. 10.1371/journal.pgen.0020190 PMC1713260 17194218

[ece36809-bib-0069] Petit, R. J. , el Mousadik, A. , & Pons, O. (2008). Identifying Populations for Conservation on the Basis of Genetic Markers. Conservation Biology, 12(4), 844–855. 10.1111/j.1523-1739.1998.96489.x

[ece36809-bib-0070] Pritchard, J. K. , Stephens M., Donnelly P., (2000). Inference of population structure using multilocus genotype data. Genetics, 155, 945–959. 10835412 10.1093/genetics/155.2.945 PMC1461096

[ece36809-bib-0071] Prunier, J. G. , Kaufmann, B. , Fenet, S. , Picard, D. , Pompanon, F. , Joly, P. , & Lena, J. P. (2013). Optimizing the trade‐off between spatial and genetic sampling efforts in patchy populations: Towards a better assessment of functional connectivity using an individual‐based sampling scheme. Molecular Ecology, 22(22), 5516–5530. 10.1111/mec.12499 24118539

[ece36809-bib-0072] R Core Team . (2019). R: A language and environment for statistical computing. R Foundation for Statistical Computing.

[ece36809-bib-0073] Ramalho, C. E. , Ottewell, K. M. , Chambers, B. K. , Yates, C. J. , Wilson, B. A. , Bencini, R. , & Barrett, G. (2018). Demographic and genetic viability of a medium‐sized ground‐dwelling mammal in a fire‐prone, rapidly urbanizing landscape. PLoS One, 13(2). 10.1371/journal.pone.0191190 PMC5812552 29444118

[ece36809-bib-0074] Raymond, M. , & Rousset, F. (1995). GENEPOP (Version 1.2): Population‐genetics software for exact tests and ecumenicism. Journal of Heredity, 86, 248–249. 10.1093/oxfordjournals.jhered.a111573

[ece36809-bib-0075] Rocha, E. C. , Brito, D. , Silva, P. M. , Silva, J. , Bernardo, P. V. S. , & Juen, L. (2018). Effects of habitat fragmentation on the persistence of medium and large mammal species in the Brazilian Savanna of Goiás State. Biota Neotropica, 18(3). 10.1590/1676-0611-bn-2017-0483

[ece36809-bib-0076] Safner, T. , Miller, M.P. , Mcrae, B.H. , Fortin, M.J. , Manel, S. (2011). Comparison of Bayesian clustering and edge detection methods for inferring boundaries in landscape genetics. International Journal of Molecular Sciences, 12, 865–889. 21541031 10.3390/ijms12020865 PMC3083678

[ece36809-bib-0077] Saitou, N. ; Nei, M. (1987) The neighbor‐joining method: a new method for reconstructing phylogenetic trees, Molecular Biology and Evolution, 4, (4), 406–425, 10.1093/oxfordjournals.molbev.a040454. 3447015

[ece36809-bib-0078] Schuelke, M. (2000). An economic method for the fluorescent labeling of PCR fragments. Nature Biotechnology, 18(2), 233–234. 10.1038/72708 10657137

[ece36809-bib-0079] Shirk, A. J. , Landguth, E. L. , & Cushman, S. A. (2017). A comparison of individual‐based genetic distance metrics for landscape genetics. Molecular Ecology Resources, 17(6), 1308–1317. 10.1111/1755-0998.12684 28449317

[ece36809-bib-0080] Silveira, L. , Henrique, F. , Rodrigues, G. , de Almeida Jácomo, A. T. , & Filho, J. A. F. D. (1999). Impact of wildfires on the megafauna of Emas National Park, Central Brazil. Oryx, 33, 108–114. 10.1046/j.1365-3008.1999.00039.x

[ece36809-bib-0081] Slatkin, M. (1993). Isolation by distance in equilibrium and non‐equilibrium populations. Evolution, 47(1), 264–279. 10.1111/j.1558-5646.1993.tb01215.x 28568097

[ece36809-bib-0082] Sokal, R. R. (1979). Testing statistical significance of geographic variation patterns. Systematic Zoology, Washington, 28(2), 227–232.

[ece36809-bib-0083] Sullivan, E. R. , Barker, C. , Powell, I. , & Ashton, P. A. (2019). Genetic diversity and connectivity in fragmented populations of *Rhinanthus minor* in two regions with contrasting land‐use. Biodiversity and Conservation, 28, 3159–3181.

[ece36809-bib-0084] Swart, M. K. J. , Ferguson, J. W. H. , Toit, R. , & Flamand, J. R. B. (1994). Substantial Genetic Variation in Southern African Black Rhinoceros (*Diceros bicomis*). Journal of Heredity, 85(4), 261–266. 10.1093/oxfordjournals.jhered.a111453 7930498

[ece36809-bib-0085] Taylor, A. C. , Sherwin, W. B. , & Wayne, R. K. (1994). Genetic variation of microsatellite loci in a bottlenecked species: The northern hairy‐nosed wombat *Lasiorhinus krefftii* . Molecular Ecology, 3(4), 277–290. 7921355 10.1111/j.1365-294x.1994.tb00068.x

[ece36809-bib-0086] Teixeira, Z. F. , Kindel, A. , Hartz, S. M. , Mitchell, S. , & Fahrig, L. (2017). When road‐kill hotspots do not indicate the best sites for road‐kill mitigation. Journal of Applied Ecology, 54(5), 1544–1551.

[ece36809-bib-0087] Templeton, A. R. , Robertson, R. J. , Brisson, J. , & Strasburg, J. (2001) Disrupting evolutionary processes: The effect of habitat fragmentation on collared lizards in the Missouri Ozarks. Proceedings of the National Academy of Sciences of USA ‐ PNAS, 98(10), 5426–5432. 10.1073/pnas.091093098 PMC33229 11344289

[ece36809-bib-0088] Théry, N. M. (2011). Conservation of natural areas in São Paulo. Estudos Avançados, 25, 175–188.

[ece36809-bib-0089] Torres‐Florez, J. P. , Hucke‐Gaete, R. , Rosenbaum, H. , & Figueroa, C. C. (2014). High genetic diversity in a small population: The case of Chilean blue whales. Ecology and Evolution, 4(8), 1398–1412. 24834336 10.1002/ece3.998 PMC4020699

[ece36809-bib-0090] Vernesi, C. , Bruford, M. W. , Bertorelle, G. , Pecchioli, E. , Rizzoli, A. , & Hauffe, H. C. (2008). Where’s the conservation in conservation genetics? Conservation Biology, 22(3), 802–804. 18336619 10.1111/j.1523-1739.2008.00911.x

[ece36809-bib-0091] Wan, Q. H. , Wu, H. , Fujihara, T. , & Fang, S. G. (2004). Which genetic marker for which conservation genetics issue? Electrophoresis, 25, 2165–2176. 15274000 10.1002/elps.200305922

[ece36809-bib-0092] Wang, J. (2017). The computer program structurefor assigning individuals to populations: Easy to use but easier to misuse. Molecular Ecology Resources. 17: 981–990. 10.1111/1755-0998.12650 28028941

[ece36809-bib-0093] Weir, B. S. , & Cockerham, C. C. (1984). Estimating F statistics for the analysis of population structure. Evolution, 38, 1358–1370. 10.2307/2408641 28563791

[ece36809-bib-0094] Weiss, M. , & Leese, F. (2016). Widely distributed and regionally isolated! Drivers of genetic structure in *Gammarus fossarum* in a human‐impacted landscape. BMC Evolutionary Biology, 16(153). 10.1186/s12862-016-0723-z PMC4966747 27473498

[ece36809-bib-0095] Wolff, J. O. , Schauber, E. M. , & Edge, W. D. (1997). Effects of habitat loss and fragmentation on the behavior and demography of Gray‐tailed voles. Biological Conservation, 11, 945–956.

[ece36809-bib-0097] Zavala‐Páramo, A. S. , Oyama, K. , Mendoza, E. , Zavala‐Páramo, M. G. , Pollinger, J. , & Smith, T. B. (2017). Genetic variability in captive individuals of the endangered species *Tapirus bairdii* in Mexico. Mexican Journal of Biodiversity, 88(2), 480–484. 10.1016/j.rmb.2017.03.002

